# Chitosan Combined with Molecular Beacon for Mir-155 Detection and Imaging in Lung Cancer

**DOI:** 10.3390/molecules190914710

**Published:** 2014-09-16

**Authors:** Hai-Zhen Zhu, Jiang-Hong An, Quan Yao, Jing Han, Xue-Tao Li, Fei-Long Jiang, Guang-Peng Chen, Li-Na Peng, Yong-Sheng Li, Jian-Guo Sun, Zheng-Tang Chen

**Affiliations:** 1 Cancer Institute of PLA, Xinqiao Hospital, Third Military Medical University, Chongqing 400037, China; E-Mails: zhuhaizhen_1234@163.com (H.-Z.Z.); 13883929713@163.com (J.H.); snowrobin2000@163.com (X.-T.L.); ilujfl@163.com (F.-L.J.); cgpfriend@163.com (G.-P.C); penglinaaini@126.com (L.-N.P.); liyongsheng_123@163.com (Y.-S.L.); 2 Department of Oncology, People’s Liberation Army 532 Hospital, Huangshan 242700, China; E-Mail: anjianghong_123@163.com; 3 Diagnosis and Treatment Center of Cancer, Chengdu Military General Hospital, Chengdu 646000, China; E-Mail: yaoquan_123@163.com

**Keywords:** lung cancer, microRNA, molecular beacon, chitosan, nanoparticles, molecular imaging

## Abstract

Lung cancer is the major cause of cancer-related deaths worldwide, thus developing effective methods for its early diagnosis is urgently needed. In recent years, microRNAs (miRNAs, miR) have been reported to play important roles in carcinogenesis and have become potential biomarkers for cancer diagnosis and treatment. Molecular beacon (MB) technology is a universal technology to detect DNA/RNA expression in living cells. As a natural polymers, chitosan (CS) nanoparticles could be used as a carrier for safe delivery of nucleic acid. In this study, we developed a probe using nanoparticles of miR-155 MB self assembled with CS (CS-miR-155 MB) to image the expression of miR-155 in cancer cells. Hybridization assay showed that the locked nucleic acid (LAN) modified miR-155 MB could target miR-155 effectively and sensitively. The miR-155 MB self-assembly with CS nanoparticles formed stable complexes at the proper weight ratio. The CS nanoparticles showed higher fluorescence intensity and transfection efficiency than the lipid-based formulation transfection agent by confocal microscopy and flow cytometry analysis. The CS-MB complexes were found to be easily synthesized and exhibited strong enzymatic stability, efficient cellular uptake, high target selectivity and biocompatibility. The CS-MB complexes can also be applied in other cancers just by simply changing for a targeted miRNA highly expressed in those cancer cells. Therefore, it is a promising vehicle used for detecting miRNA expression in living cells.

## 1. Introduction

Lung cancer is the major cause of cancer-related deaths, among which non-small-cell lung cancer (NSCLC) accounts for nearly 80% of the fatalities. Usually, lung cancer cannot be diagnosed until its late stages [1], therefore, it is important to identify and validate diagnostic and prognostic biomarkers to improve the clinical outcome of lung cancer treatment [2]. Recently, microRNAs have exhibited great potential for diagnosing and treating diseases as a novel type of biomarkers and therapeutic targets, and many chemical tools have been developed for microRNA detection, regulation, and microRNA-related biomedical applications [3,4]. MicroRNAs are known to be closely related to diverse cellular activities [5]. In recent years, miR-155 was found to have potential diagnostic and prognostic value, and was reported to be associated with esophageal cancer, lymphoma, colorectal cancer, leukemia, breast cancer and other carcinomas, especially lung cancer [6,7]. As an oncogene, miR-155 contributes to tumorigenesis, proliferation, invasion and angiogenesis [8,9,10]. Aberrant expression of miR-155 in lung cancer cells may be a potential diagnostic biomarker for early detection of the disease [11]. Tumor-initiating cells (TICs) are considered to be responsible for tumorigenesis, metastasis, and recurrence. They can provide clues for the early diagnosis and represent a potential treatment target. Furthermore, miRNAs are proved to be one of the determinants of the stemness [12]. In our previous study, we also found the expression of miR-155 expression was higher in TICs than in A549 lung cancer cells [13].

Since miRNAs are short in length, have low expression levels and have similar nucleotide sequences, techniques used to detect them need to be improved. Polymerase chain reaction (PCR), microarray analysis and Northern blotting are currently available techniques, but they are time-consuming, costly and unable to analyze *in situ* or intracellular miRNAs [14,15]. The molecular beacon technology can specifically identify single base-pair mismatches, and thus has a very high specificity [16]. When the complementary target DNA/RNA sequences are present, the MB spontaneously undergoes a conformational change with the stem opening and this leads to a fluorescence restoration. MB technology has been extensively applied to detecting DNA/RNA hybridization, rapidly analyzing gene mutation and DNA/RNA biosensors, and so on [17]. Therefore, MB may be a very simple, direct, and useful technology for detecting miRNAs in living cells [18]. As negatively charged hydrophilic molecules, nucleic acid probes cannot shuttle the cell membrane freely, so other tools or materials are needed, such as transfection reagents and nanomaterials that can facilitate cellular transfection. Moreover, nucleic acid probes may become unstable after cellular delivery due to endogenous nuclease digestion [19], so its critical to find suitable materials for efficient cellular delivery and maintenance of stability after cellular delivery.

At present, nanotechnology can potentially be used for cancer detection, diagnosis and treatment [20]. Nanoparticles of chitosan, a natural polymer, can be used for nucleic acid delivery due to their low immunogenicity and low toxicity, with high biocompatibility and positive charge. Positively charged CS nanoparticles could bind to negatively charged DNA or small interfering RNA (siRNA) and condense them into core-shell nanoparticles, thus could be used for safely delivering gene materials, such as plasmid DNA, oligonucleotides and siRNA [21].

Like DNA or siRNA, miRNAs are also short gene sequences. The polymer CS has been extensively applied to the delivery of nucleic acids, so we inferred that CS nanoparticles might be an ideal biomaterial carrier for MB delivery and miRNA detection in living cells. This study aimed to deliver MB probes to the cytoplasm using CS nanoparticles, and meanwhile facilitate MB probes to target miR-155 molecules MB probes in lung cancer cells.

## 2. Results and Discussion

### 2.1. Synthesis Optimum CS-MB Nanoparticals Complexes

**Figure 1:**
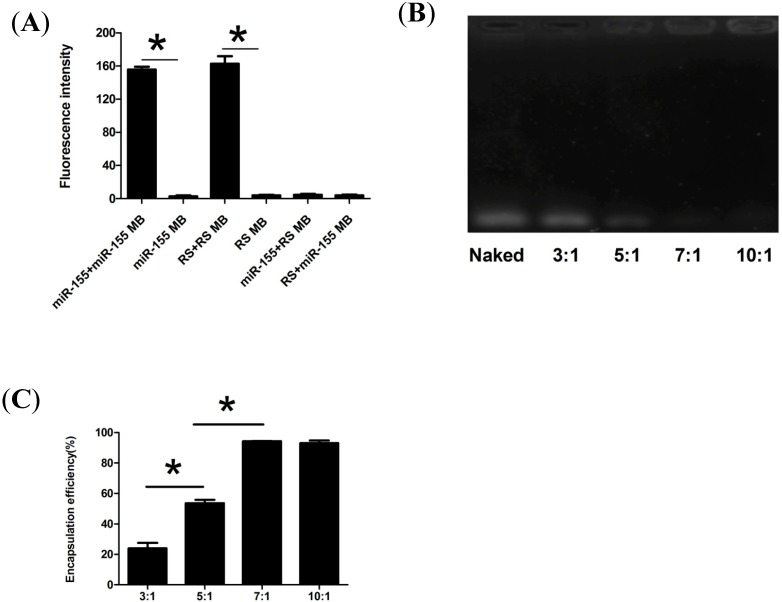
Synthesis of CS-MB nanoparticles complexes. (**A**) Hybridization experiments for the MBs. The fluorescence intensity of miR-155+miR-155 MB group, miR-155 MB group, RS+RS MB group, RS MB group, miR-155+RS MB group and RS+miR-155 MB group. (n = 4) (* *p* < 0.05). (**B**) Agarose gel electrophoresis analysis of CS-miR-155 MB complexes. Lane 1: naked miR-155 MB, lane 2–6: the different Wcs/W_miR-155 MB_ weight ratios ranging from 3:1 to 10:1. (**C**) The encapsulation efficiency of different Wcs/W_miR-155 MB_ weight ratios ranging from 3:1 to 10:1. (n = 3). (* *p* < 0.05).

The miR-155 MB and random sequence (RS) MB were assayed in both the presence and absence of their complementary designed target sequence to detect their sensitivity and specificity. The RS MB complementary to no known gene sequence was used as a negative control. Hybridization assay showed that the fluorescence intensity in the miR-155+miR-155 MB group was 55 times higher than that in the only miR-155 MB group ,and the fluorescence intensity in the RS+RS MB group was 41 times higher than that in the only RS MB group (*p* < 0.05). In addition, in the presence of the non-complementary sequences, the miR-155+RS MB group and RS+miR-155 MB group showed the same fluorescence intensity as the only RS MB group and miR-155 MB group with no significant difference among these groups (Figure 1A). The designed MB retained the stem-loop structure functionality and resulted in very low fluorescence signals when exiting on its own or other non-complementary sequences. However, in the presence of the targeted sequence, the MB efficiently separated the fluorophore from the quencher and released a high-intensity fluorescent signal, demonstrating the successful design of the MB probe and indicating that functional MB targeted reciprocal sequences correctly, effectively and sensitively.

However, the delivery of bare MB is commonly restricted by poor cellular uptake and rapid degradation. Of the carriers studied, cationic polymers condense nucleic acids efficiently through electrostatic association and arrange them into complexes [22]. In addition, they facilitate the complexes to avoid endosomal degradation via proton sponge effect and move into the cytoplasm [23]. CS nanoparticles have been considered a perfect candidate that can be applied in biomedical events such as tissue engineering, drug delivery and gene delivery [24]. At present, CS-based carriers have attracted more and more attention as a reliable system for delivering genes [14], so we concluded that CS nanoparticles could be used as an ideal biomaterial carrier for MB delivery, while simultaneously detecting the miRNA in living cells. The basic method for synthesizing CS-DNA complexes is to directly mix the positively charged CS with the negatively charged nucleic acid [25,26,27]. To obtain stable CS-MB complexes, different weight ratios of CS nanoparticles/miR-155 MB were mixed in phosphate buffered saline (PBS) buffer (pH = 6.0). A DNA retardation assay demonstrated that CS nanoparticles could not form stable complexes with miR-155 MB at the weight ratios between 3:1 and 5:1. When the weight ratio increased, CS could wrap miR-155 MB and form stable complexes as seen by the band disappearance, at the weight ratiod of 7:1 and 10:1 (Figure 1B). The encapsulation efficiency of miR-155 MB was also enhanced with the increase of weight ratio, being 94.3% and 93.02% at the ratios of 7:1 and 10:1, respectively. There was a significant difference in the encapsulation efficiency at the weight ratios ranging from 3:1 to 7:1 (*p* < 0.05). However, no significant difference was observed at the weight ratios between 7:1 and 10:1 (Figure 1C). In particular, these results (Figure 1B,C) demonstrated that the miR-155 MB self-assembly with CS could form stable complexes in PBS buffer at the weight ratios of 7:1 and 10:1, and the 7:1 weight ratio was adopted to perform the following experiments.

### 2.2. Physicochemical Characteristics of CS-MB Nanoparticle Complexes

**Figure 2:**
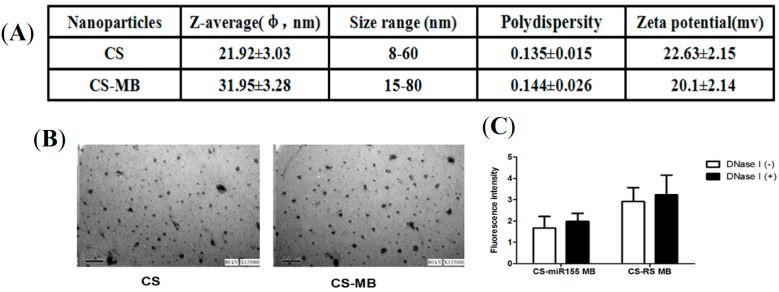
Physicochemical characteristics of CS-MB nanoparticals complexes. (**A**) The mean size, size range, polydispersity and zeta potential of CS and CS-MB. (n = 3). (**B**) TEM images of CS and CS-MB. Scale bar = 100 nm. (**C**) Fluorescence intensity of CS-MB with or without DNase I (n = 5).

Particle size is a major variable in the level of cell and tissue uptake of nanostructures. Assessment of zeta potential is also important in nanoparticle studies, as it may affect both particle stability and mucoadhesion [28]. The sizes of the CS nanoparticles and CS-MB complexes detected by the Malvern instrument were 8–60 nm and 15–80 nm, with an average of 21.92 ± 3.03 nm and 31.95 ± 3.28 nm, respectively. The mean zeta potentials of CS and CS-MB were + 22.63 ± 2.175 mV and + 20.1 ± 2.14 mV, respectively (Figure 2A).Transmission electron microscopy (TEM) revealed that CS/CS-MB had a relatively spherical shape (Figure 2B) with sizes of about 10–80 nm. The positively charged zeta potential and nanoscale-size nanoparticles were thus suitable for cell uptake.

One of the drawbacks of conventional MBs is that they tend to be digested by nuclease enzyme, which limits their application in living cells. Therefore, the integrity of CS-MB complexes in the presence of deoxyribonuclease I (DNase I) was detected. The DNase I digestion assay revealed a much slower increase in fluorescence signal for CS-MB with DNase I, with no significant difference in both the presence and absence of DNase I (Figure 2C), indicating that the stability was enhanced because of the resistance of CS nanoparticles and the bases modified by LNAs to enzymatic digestion. Oligonucleotides modified by LNAs are single-stranded bicyclic RNA analogues with a methylene bridge that links the 2' oxygen with the 4' carbon of the ribose [29]. The advantages of LNAs are that they have resistance against exo- and endonucleases and can irreversibly bind to genes [30]. LNAs can improve conformational stability, resistance against degradation and thermostability, which can produce low background signal and efficient target hybridization. At present, LNAs are used in many researches in biological synthesis, including siRNAs, aptamers, antagomirs and microRNAs [31]. CS nanoparticles can also encapsulate and protecte DNA from degradation. We found in this experiment that the CS nanoparticles and LNAs were resistant to DNase I degradation, which means that they can be used to deliver MB to cells.

### 2.3. Fluorescence Imaging and Detecting in Viable Cells

To determine whether the CS-MB complexes could detect the level of miRNA expression in viable cells, their ability to shuttle the cell membrane and detect target microRNAs was further assessed. A human prostate cancer cell line (PC-3) was used as the positive control based on our previous research [32].

**Figure 3:**
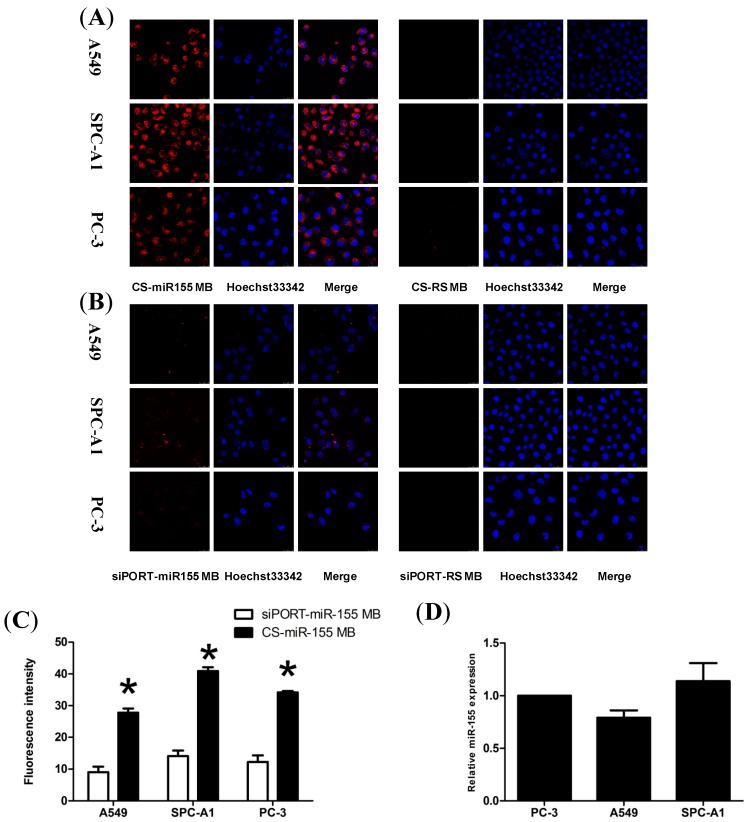
Fluorescence imaging and detecting in viable cells. (**A**,**B**) Confocal microscopy imaging of the three cells transfected with CS-miR155 MB and CS-RS MB complexes or transfected by the commercial siPORT transfection agent. RS MB was used as a negative control. All cell nucleuses were stained by Hoechst33342 (blue). Scale bar = 25 μm. (**C**) Fluorescence intensity of Cy5 was measured after incubation with CS-miR155 MB or siPORT-miR155 MB. * *p* < 0.05. (**D**) The relative miR-155 expression levels were analyzed in A549, SPC-A1 and PC-3 cells using qRT-PCR.

Confocal microscopy results showed that when the three cell lines (A549, SPC-A1 and PC-3) were treated with CS-miR-155 MB nanoparticles, a higher red fluorescence signal was observed in most of the cells because of the efficient MB delivery and targeting of the mature miR-155. The most red fluorescence was located in the cytoplasm, and the fluorescence signal was not detected when treated with CS-RSMB nanoparticles (Figure 3A). These results indicated the CS-miR-155 MB complexes could detect and image the miR-155 if it existed. In the absence of target sequence, the RS MB retained the stem-loop structure functionality and did not generate fluorescence signals. The commercial siPORT NeoFX transfection agent is a lipid-based formulation, which was also used to transfect the MBs. Confocal microscopy showed low fluorescence intensity in the siPORT-miR-155 MB group, and no red fluorescence signals in the siPORT-RS MB group (Figure 3B).

In addition, the relative expression level of miR155 was quantified by calculating the average fluorescence intensity in the cells. The CS nanoparticles showed higher fluorescence intensity than the siPORT transfection agent (Figure 3C). A significant difference was observed between the CS-miR-155 MB group and siPORT-miR-155 MB group (*p* < 0.05). The average fluorescence intensity of the CS-miR-155 MB group was found to be consistent with the level of miR-155 expression determined in our preliminary work using real-time reverse-transcriptase polymerase chain reaction (qRT-PCR) [32] (Figure 3D). The qRT-PCR result showed that the miR-155 expression levels of A549 and SPC-A1 were 0.79 and 1.14 times higher than that of the PC-3, respectively. The average fluorescence intensity result demonstrated that the miR-155 expression levels of A549 and SPC-A1 were 0.81 and 1.19 times higher than that of the PC-3, respectively. They have the same miR-155 expression pattern. The CS-MB complexes were stable in PBS buffer and under physiological conditions for at least 72 h.

### 2.4. Flow Cytometry Analysis and Cytotoxicity Assay

**Figure 4:**
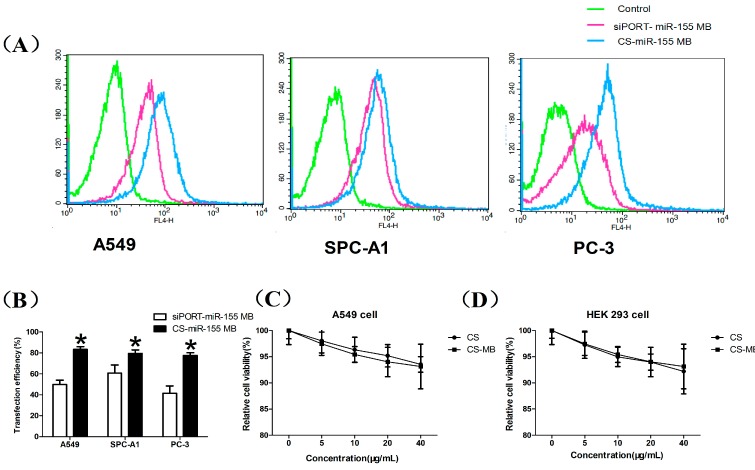
Flow cytometry analysis and cytotoxicity of CS and CS-MB. (**A**,**B**) Graphs by flow cytometry analysis and transfection efficiency of the three cell lines transfected with siPORT-miR-155 MB and CS-miR-155 MB (n = 3). * *p* < 0.05. (**C**,**D**) Cytotoxicity of CS and CS-MB to A549 cells and HEK 293 cells (n = 5).

The miR-155 MB transfection efficiency by CS nanoparticles and siPORT lipid was also detected by flow cytometry which showed that the CS nanoparticles had high transfection efficiency in the three cell lines (Figure 4A). The CS nanoparticles and siPORT transfection efficiency was 75%–85% and 40%–60% respectively, according to the flow cytometry analysis (Figure 4B). These results suggested that the CS nanoparticles had high biological stability and high transfection efficiency, making them an ideal biomaterial for *in vitro* cell imaging and detecting the relative expression of miRNA in living cells. However, the reliable quantification of miRNA expression and tracking of gene expression changes in cells need to be further studied according to previous research [18,33]. Using MB and nanotechnology, we successfully delivered MBs to detect the intracellular miR-155 highly expressed in living cancer cells. Current research suggestd that biosensor-based techniques for miRNA assay will complement amplification-based molecular techniques due to the advantages of amplification-free detection, multiplexing capability, minimal sample preparation requirement and will realize sensitive detection of microRNAs of low abundance [34]. Moreover, the incubation period in our research is two hours while it is only ten minutes or one hour in other research using streptolysin-O-based membrane permeabilization or magnetic nanoparticles [33,35]. The flow cytometry analysis showed a transfection efficiency of 75%–85%, which could be optimized in further studies.

We also assessed the cytotoxicity of the CS nanoparticles and CS-MB complexes. As shown in Figure 4C,D, the minimal cell viability was around 90% tested from 5 μg/mL to 40 μg/mL of CS nanoparticles and CS-MB complexes. These data demonstrated a low cytotoxicity of CS nanoparticles and CS-MB complexes, indicating they could be used as nanoprobes for detecting miRNA gene expression in living cells.

## 3. Experimental Section

### 3.1. Hybridization Assay

MiR-155 MB (5'-Cy5-C+CAGCG-ACC+CCT+ATCA+CGAT+TAGCATTAA-CGCT+GG-BHQ3-3') was designed according to the hsa-miR-155 sequences. A random sequence MB (RS MB: 5'-Cy5-C+CAGCG-AC+GCCA+ATG+ACC+TTA+AGCATTAA-CGCT+GG-BHQ3-3') was used as a negative control. The MB had a Cy5-molecule attached to the 5'-end and a black hole quencher-3 (BHQ3) attached to the 3'-end. The underlining bases were the ones added to form a stem, and the +N represented the LNAs modified bases. All the synthetic oligodeoxynucleotides were purchased from Sangon Company (Shanghai, China). The purpose of this experiment was to prove if the designed MB probes could target the complementary sequences correctly and produce high intensity fluorescent signals. A hybridization reaction was performed by adding both the MBs and the complementary sequences into 20 nM Tris-HCL buffer containing 5 mM MgCl_2_ and 50 mM KCl in a black 96-well microplate. The final concentration of target sequences and MBs were 1000 nM and 100 nM respectively, for the purpose of complete reaction. The miR-155 sequence and RS MB or RS sequence and miR-155 MB reaction were also performed at the same concentration (100 nM). The black 96-well plate was incubated until the signal reached a plateau at 37 °C in the dark. The fluorescence levels were monitored by measuring the fluorescence signals of Cy5 wavelengths (excitation 649 nm/emission 670 nm) using the Varioskan Flash (Thermo, Waltham, MA, USA).

### 3.2. DNA Retardation Assay

Water-soluble CS nanoparticles with a molecular weight (MW) of 50kDa and with >90% degree of deacetylation were kindly provided by Guanghan Hengyu Company (Sichuan, China). To obtain stable CS-DNA complexes, different weights of CS nanoparticles and 2 μg miR-155 MB solution were mixed in PBS buffer (Boster, Wuhan, China) that was adjusted to pH 6.0 with acetic acid. The weight ratios ranged from 3:1 to 10:1. In all cases, the mixtures were vortex shocked for 60 s and then formed at room temperature for 30 min. These samples were analyzed in the presence or absence of miR-155 MB with nanoparticles. The CS-miR-155 MB complexes were observed by the retardation bands on agarose gel electrophoresis and visualized with a ChemilmagerTM 5500 system (Alpha Innotech Corp., Knoxville, TN, USA).

### 3.3. Encapsulation of Mir-155MB

Different weight ratios (3:1, 5:1, 7:1 and 10:1) of CS nanoparticles and miR-155 MB (100 ng) complexes were mixed according to the method described earlier in the present study. The solution was centrifuged at 20,000 rpm at room temperature for 60 min and the supernatant was collected to determine the amount of free miR-155 MB. The unbound mir-155 MB content was quantified by UV spectrophotometry. The encapsulation efficiency (EE%) was determined using the following formula:

EE% = (Total sum of miR-155 MB − Free sum of miR-155 MB)/Total sum of miR-155 MB × 100%.

### 3.4. Characterizations of Size, Zeta Potential and Morphology

CS- miR-155 MB complexes were prepared in PBS at the weight ratio of 7:1 as described. The nanoparticle sizes and zeta potential of CS nanoparticles and CS-miR-155 MB complexes were detected by a Malvern Zetasizer Nano instrument (Malvern 3000HS, Worcestershire, UK) at 25 °C. The morphology of CS nanoparticles and CS-miR-155 MB complexes were imaged using high-resolution TEM (Philips TECNAI 10, Amsterdam, Holland). The samples were deposited on a carbon-coated copper grid and were dried for 10 min. After that, the samples were stained with neutral 2% aqueous phosphotungstic acid for 60 s.

### 3.5. DNase I Assay

The integrity of condensed and encapsulated MBs in CS nanoparticles was investigated by DNase I (Beyotime, Shanghai, China) digestion assay. The CS-MB complexes were incubated with DNase I (1 U/μL) in reaction buffer at 37 °C for 30 min and then the reaction was stopped by adding EDTA at 65 °C for 10 min. The final concentration of MB was 100 nM, and the fluorescence signals were monitored.

### 3.6. Confocal Microscopy

The human NSCLC and prostate cancer cell lines (A549 and PC-3) were purchased from the ATCC (Manassas, VA, USA). The human NSCLC cell line SPC-A1 and HEK 293 cell line were purchased from the Shanghai Institute of Cellular Biology. HEK 293 and other cell lines were cultured in high glucose DMEM medium or PRMI 1640 medium (Hyclone, Waltham, MA, USA) supplemented with 10% fetal calf serum, respectively. The ability of CS-MB complexes to permeate the cell membrane and detect target miRNA was further investigated. All three cell lines were seeded in a dish (glass bottom cell culture dish, especial for confocal microscopy) in an amount of 1 × 10^4^ cells for confocal microscopy. After 24 h, the culture medium were decanted and washed three times with sterile PBS for 3 min each time and then replaced with 300 μL Opti-MEM^®^ I Reduced Serum Medium (Life Technologies, Waltham, MA, USA) mixture which contains CS-MB complexes. The final concentration of CS-MB complexes was 200 nM. After being incubated at 37 °C in a CO_2_ incubator for 2 h, cells were washed three times for 3 min each time with sterile PBS. Then, the living cells were stained with 200ul of Hoechst 33342 (Beyotime, Shanghai, China), incubated at 37 °C for 20 min, and washed three times with PBS for 3 min again. After Hoechst 33342 staining, the fluorescence images were obtained using a confocal microscope (Leica TCS confocal microscope, Leica, Wetzlar, Germany), and then 1 mL RIPA buffer (Beyotime) was added for lysing the cells in the dish. The liquid suspension was plated into 6 wells of the black 96-well plate with 100 μL per well and the fluorescence intensity was measured by Varioskan Flash. As the negative control, the three cell lines treated with CS-RSMB (200 nM) were prepared and monitored in the same procedure. The fluorescence intensity was detected to show the expression level of miR-155 gene in the cells. We also used the commercial siPORT NeoFX transfection agent (Ambion, Waltham, MA, USA) to transfect miR-155 MB and RSMB with the method adopted in our previous research [32], and the fluorescence intensity was measured by Varioskan Flash using the same procedure.

### 3.7. Flow Cytometry and MTT Assay

The transfection efficiency of CS nanoparticles and siPORT lipid was also detected by flow cytometry (BD FACSCalibur, Becton, Dickinson and Company, Franklin Lakes, NJ, USA). Using the same method, after being transferred by the CS or siPORT combined with miR-155 MB or RSMB, the cells were digested and collected by adding 1 mL 0.05% trypsin solution in the dish, and then the collected cells were analyzed by the flow cytometry. The cytotoxicity of CS nanoparticles and CS-MB complexes to A549 and HEK 293 cells with concentration of 5, 10, 20 and 40 μg/mL was evaluated by MTT assay. After being incubated with different concentration of CS nanoparticles or CS-MB complexes for 24 h, the absorbance was measured using the multifunctional microplate reader at 490 nm. The relative cell viability (%) was calculated by (A_test_/A_control_) × 100. The percent cell viability of the control cells was viewed as 100%.

### 3.8. Statistical Analysis

The two groups were compared by an unpaired Student’s *t*-test. One-way analysis of variance was used in multiple group by SPSS version 17.0. Data were presented as the means ± standard deviations. A *p* value less than 0.05 was considered statistically significant.

## 4. Conclusions

At present, the investigation of miRNAs as biomarkers for improving the diagnosis of NSCLC is just at the onset. MBs have the potential to serve as powerful tools for quantifying miRNA expression. Moreover, different MBs could be designed and applied as targeted diagnostic probes to detect other highly expressed miRNAs in many different cancers or TICs. Nanocarriers preferentially accumulate in tumor tissues. Our study demonstrates that it is safe and convenient to prepare CS combined with MB, which requires no complicated or time-consuming chemical conjugation steps. Hence this promising way for detecting miRNA expression in living cells may provide a new avenue for cancer diagnosis in the future.
